# Independent and Combined Associations between Physical Activity and Sedentary Time with Sleep Quality among Chinese College Students

**DOI:** 10.3390/ijerph19116697

**Published:** 2022-05-30

**Authors:** Dan Li, Xianxiong Li

**Affiliations:** School of Physical Education, Hunan Normal University, Changsha 410012, China; lidan97@hunnu.edu.cn

**Keywords:** sleep quality, physical activity, sedentary time, Chinese college students

## Abstract

Objective: To investigate the independent and combined associations between physical activity and sedentary time with sleep quality among Chinese college students. Methods: A cross-sectional study was conducted among Chinese college students (N = 2347; M age = 20 years; 67.1% students were female). We used the International Physical Activity Questionnaire—Short Form, the Adolescent Sedentary Activity Questionnaire, and the Pittsburgh Sleep Quality Index to assess the subjects’ physical activity level, sedentary time, and sleep quality, respectively. Analyses were conducted using a multivariate logistic regression model. Result: Of the total participants, 48.6% had poor sleep quality and 10% were at low physical activity levels, and the mean (±SD) sedentary time was 5.33 ± 2.34 h/day. No significant association was found between physical activity and sleep quality (OR = 1.27, 95% CI: 0.95~1.70) among Chinese college students after adjustment for potential confounders. Sedentary time was significantly positively associated with poor sleep quality (OR = 1.37, 95% CI: 1.14~1.65). The risks for those with a low physical activity level and high sedentary time, and a moderate physical activity level and high sedentary time were 2.78 (OR = 2.78, 95% CI: 1.61~4.80) and 1.49 (OR = 1.49, 95% CI: 1.13~1.95) times higher, respectively, than those with a high physical activity level and low sedentary time. Conclusion: Among Chinese college students, high sedentary time was significantly negatively associated with sleep quality. A low physical activity level was insignificantly associated with sleep quality. A moderate physical activity level and high sedentary time, and a low physical activity level and high sedentary time were interactively associated with increased risks of poor sleep quality, respectively.

## 1. Introduction

Sleep is an essential part of everyone’s lives [1]. Sufficient sleep is a prerequisite to maintaining a well-balanced lifestyle [2]. Poor sleep quality can cause diseases, such as obesity [3], diabetes [4], cancer [5], and metabolic syndrome [6]. However, because of academics and peer pressure, the proportion of poor sleep quality among college students is on the rise [7]. Studies have highlighted that sleep problems among college students increased dramatically from approximately 27% in 1982 to approximately 68% in the last decade [8]. Indeed, college students who are transitioning from adolescence to adulthood and trying to earn academic degrees usually confront numerous new challenges that can increase the prevalence of poor sleep quality. Hence, studying sleep quality among college students makes sense.

Sleep quality is affected by various factors, and one of them is physical activity [9]. Several previous studies have found that sleep quality was better for those students who felt they performed more physical activity than those who felt they performed less physical activity [10]. After using an objective instrument for measuring physical activity, Loprinzi et al. [11] discovered that regular engagement in physical activity has a small favorable impact on total sleep duration and sleep quality. A meta-analysis reported that moderate-intensity physical activity is more effective in improving sleep quality than vigorous-intensity physical activity [12]. Furthermore, the World Health Organization suggested that adults aged 18–64 years should participate in at least 150 min of moderate-intensity physical activity or at least 75 min of vigorous-intensity physical activity per week [13]. Despite those recommendations, the latest survey showed that the vast majority of college students fail to meet the recommended amount of physical activity [14]. Therefore, the relationship between physical activity and sleep quality among college students cannot be ignored.

Furthermore, fewer studies have revealed that prolonged sedentary time is an emerging influential factor related to poor sleep quality [15]. Meanwhile, some studies have found that the duration of subsequent nighttime sleep was shorter when daytime sedentary time increased [16], and interventions that focus on regularly interrupting sedentary time could improve the sleep duration of young people [17]. However, some studies using accelerometers have reported that the average daily sedentary time ranged from 8.23 to 13.03 h per day [18,19,20,21,22], while another study found that the sedentary time of college students is increasing [23]. Consequently, this increasing trend of sedentary time among college students may be related to sleep quality.

Recently, a variety of studies have attempted to understand how the combination of physical activity and sedentary time causes certain health problems. Accordingly, studies reported that adolescents with a combination of a low physical activity level and a high sedentary time had a higher risk of obesity than those with a combination of a high physical activity level and a low sedentary time [24], and older adults with a combination of sufficient physical activity and a low sedentary time had a lower chance of mortality than those with a combination of insufficient physical activity and a high sedentary time [25]. However, to the best of our knowledge, few studies on the combined association between physical activity and sedentary time with sleep quality among Chinese college students have been performed.

Thus, our study was designed to investigate the independent and interactive association between physical activity and sedentary time with sleep quality among Chinese college students. The significant results will help to fill current gaps in the literature.

## 2. Materials and Methods

### 2.1. Participants

A cross-sectional survey was conducted in October 2020 among college students in grades 1–4 at a university in Hunan Province, China. The study was approved by the ethics committee of Hunan Normal University, and informed consent was obtained from all participants. Self-reported data regarding the gender, age, region, height, weight, as well as physical activity, sedentary time, and sleep quality were obtained using an electronic questionnaire. The questionnaire, which was completed in the classroom, took approximately 10–15 min to complete. Finally, a total of 2347 college students were enrolled in this study.

### 2.2. Measures

#### 2.2.1. Physical Activity

The International Physical Activity Questionnaire—Short Form (IPAQ-SF), which has been verified and suggested as a useful tool [26], was used to assess the physical activity of college students. The IPAQ-SF was designed to measure the frequency and duration of their walking, moderate-intensity, and vigorous-intensity physical activities over the previous 7 days. According to the guideline criteria [27], the college students’ physical activity levels could be categorized as low, moderate, or high. In our study, the internal consistency of the IPAQ-SF was found to be good (Cronbach’s α = 0.752).

#### 2.2.2. Sedentary Time

The Adolescent Sedentary Activity Questionnaire (ASAQ), which has great reliability in measuring sedentary behaviors in adolescents [28], was used to investigate the sedentary time of college students. The questionnaire consisted of 12 questions, and sedentary behavior was divided into five categories: video, transportation, culture, education, and social. Mean daily sedentary time = (sum of daily sedentary time in all categories on weekdays × 5 + sum of daily sedentary time in all categories on weekends × 2)/7. Based on the literature recommendations [29], a sedentary time of ≥6 h/day was defined as a high sedentary time. In this study, the internal consistency of the ASAQ was acceptable (Cronbach’s α = 0.703).

#### 2.2.3. Sleep Quality

The Pittsburgh Sleep Quality Index (PSQI) was used to measure the sleep quality of college students. The PSQI consisted of 19 self-rated items and 5 additional items, with the options “very good” to “very poor” scoring 0–3, respectively. The total PSQI score ranges from 0 to 21; higher scores indicate a worse quality of sleep [30]. According to the literature recommendation [31], if the PQSI total score was more than 5, it was considered “poor sleep quality”. It has been shown that the Chinese version of the PSQI has high reliability and validity [32], and the internal consistency of the PSQI in the present study was strong (Cronbach’s α = 0.831).

### 2.3. Statistical Analysis

All analyses were carried out using the Statistical Package for the Social Sciences (SPSS), version 26. Means (standard deviation, SD) were adopted to describe the measurement data and numbers (percentages, %) to describe the categorical data. A multivariate logistic regression model was used to explore the independent and combined associations between physical activity and sedentary time with sleep quality among college students. A *p* < 0.05 was considered significant for all tests.

## 3. Results

### 3.1. Student Characteristics

Table 1 presents the Chinese college students’ characteristics. Approximately half of the participants in this study were female (67.1%), and the mean age was 20 (SD: 1.19) years. Moreover, 42.2% reported a high physical activity level, while 74.1% reported a low sedentary time. The overall prevalence of poor sleep quality was 48.6%.

### 3.2. Independent Associations between Physical Activity and Sedentary Time with Sleep Quality among Chinese College Students

As shown in Table 2, compared to a high physical activity level, a low physical activity level was independently associated with a significantly higher risk of poor sleep quality (OR = 1.37, 95% CI: 1.03~1.83), however, these correlations were not statistically significant after controlling for the variables of age, gender, region, and body mass index. As shown in Table 3, compared to a low sedentary time, a high sedentary time was independently associated with a significantly higher risk of poor sleep quality (OR = 1.37, 95% CI: 1.14~1.65), and these correlations were maintained after adjusting for the variables of age, gender, region, and body mass index.

### 3.3. Combined Associations between Physical Activity and Sedentary Time with Sleep Quality among Chinese College Students

The combined associations between physical activity and sedentary time with poor sleep quality are shown in Table 4. Among the Chinese college students, we found significant associations between physical activity and sedentary time with poor sleep quality (*p* < 0.05). The poor sleep quality risks for those with a low physical activity level and high sedentary time, and a moderate physical activity level and high sedentary time were 2.78 (OR = 2.78, 95% CI: 1.61~4.80) and 1.49 (OR = 1.49, 95% CI: 1.13~1.95) times higher, respectively, than the risk for those with a high physical activity level and low sedentary time. Furthermore, these associations remained significant when controlling for the variables of age, gender, region, and body mass index.

## 4. Discussion

The survey showed that those with poor sleep quality accounted for 48.6% of the Chinese college students, which was consistent with the results of other studies [33]. Studies have shown that physical activity, health status, sleep habits, dietary habits, nutritional intake, sleep environment, study, and living environment are important factors that affect college students’ sleep [34]. The poor sleep quality among college students is mainly manifested as sleep deprivation and excessive sleep [35]. The recommendations for sleep duration indicate that young people aged 18–25 should have a sleep duration of 7–9 h/day and fall asleep before 22:00 [36].

This study found that among Chinese college students, the risk of poor sleep quality with a low physical activity level was 1.37 times higher than that with a high physical activity level; however, these correlations were not statistically significant after controlling for the variables of age, gender, region, and body mass index. Some studies also showed no significant effects of exercise or daily physical activity on sleep [37]. However, a study showed that the relative risk of regularly feeling overly sleepy during the day for people who meet the PA guidelines was 0.65 times lower than for those who did not meet the guidelines [11]. Participants with insufficient PA levels were more likely to report a higher frequency of nightmares and a greater proclivity for nightmares than those with sufficient PA levels [38]. The independent association between physical activity with sleep quality among college students has remained controversial up to now.

We also found that sedentary time was negatively associated with sleep quality among Chinese college students, a result that was similar to previous studies [39]. Recent findings have suggested that sleep quality decreased in the experimental group with increased sedentary time, compared to the control group [40]. Sedentary behavior and sleep quality have also been shown to influence each other; when sleep quality is poor, college students participate in more sedentary behavior [41]. Research exploring the neurobiological association between sedentary time and sleep quality is still in its early stages; the increased use of LED-backlit televisions and computer screens may be one potential explanation [42]. LED-backlit displays might induce a significant suppression of melatonin, disrupting the biological clock and leading to increased sleep problems in people who spend too much sedentary time in front of a screen [43]. However, a cross-sectional study showed that adolescents’ sedentary behavior is not only due to screen use [44] and that reducing sedentary time by decreasing the frequency of screen use has only a small intervention effect [45].

Our study also provides information about the combined associations between physical activity and sedentary time with sleep quality. We found that the risk of poor sleep quality for those with a low physical activity level and high sedentary time, and a moderate physical activity level and high sedentary time were 2.78 and 1.49 times higher, respectively, than the risk for those with a high physical activity level and low sedentary time among Chinese college students. This indicated that too low a level of physical activity and too high a level of sedentary time may be risk factors for sleep quality. To improve sleep quality, a combination of increasing physical activity levels and decreasing sedentary time is more effective than single-mode intervention effects [46]. A study showed that people with a high physical activity level and low sedentary time have lower rates of poor sleep quality [47] and better cardiorespiratory fitness [48] compared to people with a low physical activity level and high sedentary time.

There are some limitations to the present study. First, this study is a cross-sectional study, which can reveal only the possible association between variables and cannot determine their causal relationship. Future studies could adopt longitudinal tracking or an experimental control study design for further exploration of the associations between variables. Second, all variables were assessed using a self-reported questionnaire; objective measurement tools, such as accelerometers, can be used in the future to avoid reporting bias and enhance the persuasiveness of data. Finally, the participant sample of this study was derived only from universities in Hunan, China, and we did not match strictly for gender. In follow-up research, the sample size should be expanded.

## 5. Conclusions

The present study confirmed that, among Chinese college students, a high sedentary time was significantly negatively associated with sleep quality. A low physical activity level was insignificantly associated with sleep quality. A moderate physical activity level and high sedentary time, and a low physical activity level and high sedentary time were interactively associated with increased risks of poor sleep quality, respectively. We recommend that, for Chinese college students, it is advisable to not only improve physical activity levels but also reduce or consciously interrupt their sedentary time, thereby reducing the risk of poor sleep quality.

## Figures and Tables

**Table 1:** Characteristics of Chinese college students (n = 2347).

	N (%)	Mean	SD
Age		20	1.19
Gender			
Female	1574 (67.1)		
Male	773 (32.9)		
Region			
Rural	1375 (58.6)		
Urban	972 (41.4)		
Body mass index			
Body mass index < 18.5	498 (21.2)		
18.5 ≤ body mass index < 24	1498 (63.8)		
24 ≤ body mass index < 28	212 (9.1)		
Body mass index ≥ 28	139 (5.9)		
Physical activity level			
Low	234 (10.0)		
Moderate	1123 (47.8)		
High	990 (42.2)		
Sedentary time (h/day)		5.33	2.34
Sedentary time			
Low	608 (25.9)		
High	1739 (74.1)		
PSQI total score		5.71	3.02
Sleep quality			
Healthier sleep quality	1207 (51.4)		
Poor sleep quality	1140 (48.6)		

Values are presented as Mean ± SD or number (percentage) when appropriate.

**Table 2:** Associations between physical activity and poor sleep quality among Chinese college students.

Study Variables	Model 1 OR (95% CI)	Model 2 OR (95% CI)
Physical activity level		
High	Ref.	Ref.
Moderate	1.17 (0.98~1.38)	1.10 (0.92~1.31)
Low	1.37 (1.03~1.83) *	1.27 (0.95~1.70)
Age (years)		
Per one-unit increase		1.10 (1.03~1.18) *
Gender		
Female		Ref.
Male		0.67 (0.56~0.80) **
Region		
Rural		Ref.
Urban		1.26 (1.06~1.49)
Body mass index		
Body mass index < 18.5		Ref.
18.5 ≤ Body mass index < 24		0.94 (0.77~1.16)
24 ≤ Body mass index < 28		1.05 (0.76~1.47)
Body mass index ≥ 28		1.24 (0.84~1.82)

Model 1: examines the association of physical activity with poor sleep quality. Model 2: examines the association of physical activity with poor sleep quality adjusted for age, gender, region, and body mass index. OR: odds ratio; CI: confidence interval; Ref.: reference category. *: *p* < 0.05, **: *p* < 0.01.

**Table 3:** Associations between sedentary time and poor sleep quality among Chinese college students.

Study Variables	Model 1 OR (95% CI)	Model 2 OR (95% CI)
Sedentary time		
Low	Ref.	Ref.
High	1.37 (1.14~1.65) **	1.31 (1.09~1.58) *
Age (years)		
Per one-unit increase		1.10 (1.03~1.18) *
Gender		
Female		Ref.
Male		0.65 (0.55~0.79) **
Region		
Rural		Ref.
Urban		1.26 (1.07~1.49) *
Body mass index		
Body mass index < 18.5		Ref.
18.5 ≤ body mass index < 24		0.94 (0.77~1.16)
24 ≤ body mass index < 28		1.06 (0.76~1.47)
Body mass index ≥ 28		1.25 (0.85~1.84)

Model 1: examines the association of sedentary time with poor sleep quality. Model 2: examines the association of sedentary time with poor sleep quality adjusted for age, gender, region, and body mass index. OR: odds ratio; CI: confidence interval; Ref.: reference category. *: *p* < 0.05, **: *p* < 0.01.

**Table 4:** Combined associations between physical activity and sedentary time with poor sleep quality among college students.

Study Variables	Model 1 OR (95% CI)	Model 2 OR (95% CI)
Physical activity × Sedentary time		
High physical activity level × Low sedentary time	Ref.	Ref.
Low physical activity level × High sedentary time	2.78 (1.61~4.80) **	2.52 (1.45~4.37) **
Low physical activity level × Low sedentary time	1.18 (0.84~1.64)	1.09 (0.78~1.53)
Moderate physical activity level × High sedentary time	1.49 (1.13~1.95) *	1.34 (1.02~1.76) *
Moderate physical activity level × Low sedentary time	1.18 (0.96~1.44)	1.11 (0.91~1.36)
High physical activity level × High sedentary time	1.33 (0.99~1.77)	1.27 (0.95~1.69)
Age (years)		
Per one-unit increase		1.10 (1.03~1.18) *
Gender		
Female		Ref.
Male		0.67 (0.56~0.81) **
Region		
Rural		Ref.
Urban		1.26 (1.07~1.49) *
Body mass index		
Body mass index < 18.5		Ref.
18.5 ≤ Body mass index < 24		0.95 (0.77~1.17)
24 ≤ Body mass index < 28		1.05 (0.75~1.46)
Body mass index ≥ 28		1.25 (0.85~1.83)

Model 1: examines the combined associations between physical activity and sedentary time with poor sleep quality. Model 2: examines the combined associations between physical activity and sedentary time with poor sleep quality adjusted for age, gender, region, and body mass index. OR: odds ratio; CI: confidence interval; Ref.: reference category. *: *p* < 0.05, **: *p* < 0.01.

## Data Availability

The datasets used in this study are available from the corresponding authors on reasonable request.
